# The transit of oral premedication beyond the stomach in patients undergoing laparoscopic sleeve gastrectomy: a retrospective observational multicentre study

**DOI:** 10.1186/s12893-023-02246-6

**Published:** 2023-11-04

**Authors:** Laurence Weinberg, Nick Scurrah, Tom Neal-Williams, Wendell Zhang, Sharon Chen, Hugh Slifirski, David S. Liu, Angelica Armellini, Ahmad Aly, Anthony Clough, Dong-Kyu Lee

**Affiliations:** 1 Department of Anesthesia, Austin Health, Heidelberg, Australia; 2 Department of Critical Care, The University of Melbourne, Austin Health, Heidelberg, Australia; 3 Department of Surgery, Austin Health, University of Melbourne, Heidelberg, Australia; 4 General and Gastrointestinal Surgery Research Group, The University of Melbourne, Austin Precinct, Heidelberg, Australia; 5 Division of Cancer Surgery, Peter MacCallum Cancer Centre, Parkville, Australia; 6 Department of Surgery, Box Hill Hospital, Box Hill, Australia; 7 Melbourne Centre for Bariatric Surgery, Melbourne, Australia; 8 Department of Anesthesiology and Pain Medicine, Dongguk University Ilsan Hospital, Goyang, Republic of Korea

**Keywords:** Sleeve gastrectomy, Premedication bariatric, Obesity, Diabetes, Fast-track anesthesia

## Abstract

**Background:**

Antiemetic and analgesic oral premedications are frequently prescribed preoperatively to enhance recovery after laparoscopic sleeve gastrectomy. However, it is unknown whether these medications transit beyond the stomach or if they remain in the sleeve resection specimen, thereby negating their pharmacological effects.

**Methods:**

A retrospective cohort study was performed on patients undergoing laparoscopic sleeve gastrectomy and receiving oral premedication (slow-release tapentadol and netupitant/palonosetron) as part of enhanced recovery after bariatric surgery program. Patients were stratified into the Transit group (premedication absent in the resection specimen) and Failure-to-Transit group (premedication present in the resection specimen). Age, sex, body mass index, and presence of diabetes were compared amongst the groups. The premedication lead time (time between premedications’ administration and gastric specimen resection), and the premedication presence or absence in the specimen was evaluated.

**Results:**

One hundred consecutive patients were included in the analysis. Ninety-nine patients (99%) were morbidly obese, and 17 patients (17%) had Type 2 diabetes mellitus. One hundred patients (100%) received tapentadol and 89 patients (89%) received netupitant/palonosetron. One or more tablets were discovered in the resected specimens of 38 patients (38%). No statistically significant differences were observed between the groups regarding age, sex, diabetes, or body mass index. The median (Q1‒Q3) premedication lead time was 80 min (57.8‒140.0) in the Failure-to-Transit group and 119.5 min (85.0‒171.3) in the Transit group; *P* = 0.006. The lead time required to expect complete absorption in 80% of patients was 232 min (95%CI:180‒310).

**Conclusions:**

Preoperative oral analgesia and antiemetics did not transit beyond the stomach in 38% of patients undergoing laparoscopic sleeve gastrectomy. When given orally in combination, tapentadol and netupitant/palonosetron should be administered at least 4 h before surgery to ensure transition beyond the stomach. Future enhanced recovery after bariatric surgery guidelines may benefit from the standardization of premedication lead times to facilitate increased absorption.

**Trial registration:**

Australian and New Zealand Clinical Trials Registry; number ACTRN12623000187640; retrospective registered on 22/02/2023.

**Supplementary Information:**

The online version contains supplementary material available at 10.1186/s12893-023-02246-6.

## Introduction

Laparoscopic sleeve gastrectomy is an evidence-based and cost-effective method to achieve weight loss. Furthermore, it improves obesity-related and non-obesity-related diseases such as cardiovascular disease, type 2 diabetes and stroke, non-alcoholic fatty liver, obstructive sleep apnea, and urinary incontinence [1–6]. The implementation of enhanced recovery after bariatric surgery (ERABS) is a proven model for accelerating patient recovery without compromising the length of hospital stay, 90-day readmission, or adverse events [7, 8].

One of the guiding principles in facilitating ERABS is the prevention and management of postoperative nausea, vomiting, and pain [9]. Prophylactic antiemetic and analgesic premedication are frequently prescribed orally before surgery to enhance recovery postoperatively [10].

Laparoscopic sleeve gastrectomy involves removing approximately 65–80% of the stomach. Accordingly, it is unknown whether oral medications given preoperatively transit beyond the stomach, or if they remain in the sleeve resection specimen, thereby negating their pharmacological effects in facilitating ERABS. Therefore, we investigated if the time from when the oral premedications were administered before surgery, to the time that the sleeve resection specimen was resected (i.e., the premedication lead time) affects whether such medications are physically present or absent in sleeve resection specimen.

## Methods

### Logistics and setting

This retrospective observational study was approved by the Human Research Ethics Committee of the Austin Hospital, and the protocol was retrospectively registered in the Australian New Zealand Clinical Trials Registry (ACTRN12623000187640). The trial was conducted at the Austin Hospital, a public university teaching hospital and at Epworth Hospital, a private healthcare center, in Melbourne, Australia. The study was conducted in accordance with the STROBE guidelines for observational studies [11].

### Participants

We included patients aged 18 years or older who underwent laparoscopic sleeve gastrectomy between July 2020 and May 2022 for weight loss and received oral premedication as part of their ERABS protocol. We excluded patients undergoing non-resectional procedures including Roux-en-Y gastric bypass, single anastomosis gastric bypass, laparoscopic gastric banding, revisional bariatric surgery, patients that were on prokinetic medications, and all patients those who did not receive premedications. All surgical procedures were performed by two surgeons (AA and AC) with a high volume of practice for bariatric surgery.

### Preoperative optimization

All patients underwent an enhanced recovery after surgery program aligned with international guidelines, [12] which included a comprehensive pre-optimization program of smoking and alcohol cessation, a multidisciplinary pre-habilitation and exercise weight loss program that included a low-carbohydrate, low-calorie diet (1000–1200 kcal/day) or very low-calorie diet (800 kcal/day), and optimization of medical and psychosocial comorbidities, including sleep disordered breathing, anxiety, and depression. As part of our institution’s diabetes discovery initiative, all patients with an HbA1c level of 8.3% (67 mmol/mol) had a personalized plan for glycemia and were managed according to the American Diabetes Association consensus statement guidelines on inpatient glycaemic control [13]. Preoperatively, all patients were allowed a light meal for up to 6 h preoperatively and were encouraged to drink clear fluids for up to 2 h before their scheduled surgery.

### Preoperative premedication

Oral preoperative premedication provided for optimization of analgesia and prevention of postoperative nausea and vomiting (PONV) included two medications. The first premedication was a single, white-colored, hard capsule that contained two active substances - netupitant (300 mg) and palonosetron (0.5 mg) (Akynzeo®, Helsin Therapeutics, USA). Each Akynzeo capsule was 21.7 mm in length (capsule size “0”). The second premedication was a white coloured tapentadol slow-release tablet (6.5 × 15 mm) (Palexia®, Seqiris Pty, Victoria, Australia) available as single 50 mg or 100 mg tablets. At the discretion of the attending anaesthetist, patients were prescribed between 50 and 200 mg of tapentadol (i.e., one to four tables). The rationale for the use of tapendadol included its enhanced analgesic potency, lack of serotonin-related side effects, and lower incidence of nausea and vomiting than those of tramadol and other opioids [14, 15]. Netupitant/palonosetron was used because of its strong antiemetic properties and its long duration of action [15]. Both premedications were administered orally on the day of surgery as soon as the patient arrived in the preoperative surgical admission unit.

### Surgical technique

**Fig. 1:**
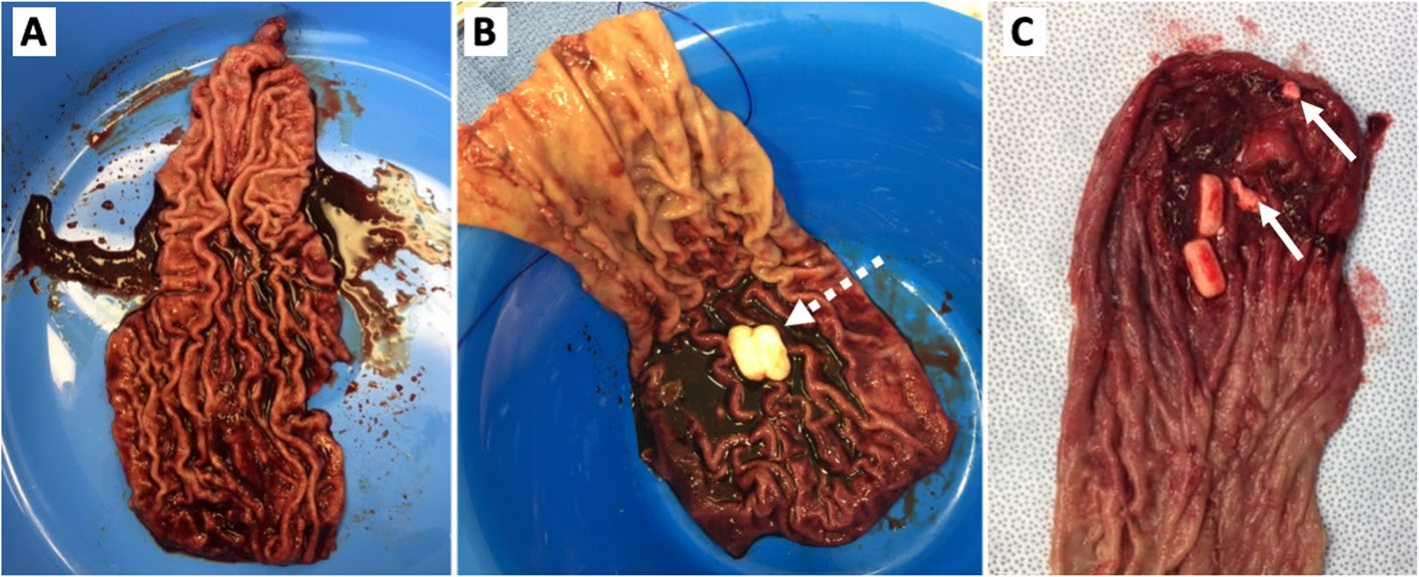
Gastric resections of three patients showing no undissolved premedication (**A**) undissolved premedication – dashed white dashed arrow (**B**) and partially dissolved premedication –white arrows (**C**) in the resected gastric specimen

Both surgeons used a similar technique. An optical trocar was used to establish pneumoperitoneum. Commencing 2–5 cm from the pylorus, the stomach was mobilized along the greater curvature to the gastroesophageal junction using the Harmonic Scalpel® (Ethicon Endo-Surgery, Inc.), exposing the left crus and fully mobilizing the fundus. Hiatus hernias were repaired when present. Commencing 2–5 cm from the pylorus, the sleeve gastrectomy was performed over a 36 French bougie with an Echelon 60 Stapler (Echelon Flex™ Endopath, Ethicon Endo-Surgery Inc.) utilizing all green loads except the most distal, which was black. Active bleeders were clipped, and 4 ml Evicel was sprayed over the staple line. Omentopexy was performed to stabilize the sleeved stomach position. The resected stomach specimen was retrieved and opened fully with scissors from the distal extent to the apex of the fundus, external to the operating field, to check for the presence of tablet medications. The sleeve resection specimen was photographed, and the number and type of tablets were recorded. An example of photographed images with and without premedications present is presented in Fig. 1. Intraoperative gastroscopy was not performed to assess if there was any medication left in the remnant stomach.

### Key outcomes

The primary outcome was the presence or absence of oral premedication in the sleeve resection specimen of patients undergoing laparoscopic sleeve gastrectomy. The secondary aims were to evaluate whether age, sex, body mass index, or diabetes mellitus were associated with the presence or absence of oral premedication in the stomach or resected gastric specimen. Finally, we evaluated the association between premedication lead time and the presence or absence of premedication in the specimen. Premedication lead time was defined as the time in minutes from when the premedication tablets were administered orally to when the gastric sleeve was resected.

### Data collected

Patient data were collected retrospectively from electronic medical records and paper-based records of local hospitals. Demographic data collected included patient age (years), weight (kg), height (cm), body mass index (kg/m^2^), and diagnosis of diabetes mellitus and other comorbidities prior to surgery.

Perioperative data collected included premedication lead time (min), dosages of oral premedication (tablets), and total amount of oral tablets administered. Intraoperative data included surgical technique, number of tablets observed in the stomach and resected gastric specimen, and their state at the time of resection (degree of visualization).

### Statistical analysis

Statistical analysis was performed using R system version 4.2.1 (R Core Team, 2022, R: A language and environment for statistical computing, Vienna, Austria). Patients were divided into two categorical groups: Failure-to-Transit and Transit groups. Continuous variables were evaluated for normality using the Shapiro-Wilk test. The Mann-Whitney test, Chi-square test, and Cochran–Armitage test for trends were used to compare demographic and preoperative information between groups. The relationship between this information and groups was evaluated using Spearman’s correlation analysis.

Kaplan-Meier survival analysis was applied to estimate the relationship between medication lead time and the presence (Failure-to-Transit group) or absent (Transit group) of premedication in the sleeve resection specimen after resection. The event was defined as the absence of medication in the surgical specimen (i.e., in the removed sleeve resection), and the presence of medication in the specimen was considered a censored case. Observation time was defined using premedication lead time. Response rates of 50% (median) and 80% complete absorption times were estimated with the corresponding 95%Cis. The proportional hazard assumption was evaluated using a log-log plot, and a Log-rank test was applied to compare the survival curves.

## Results

**Fig. 2:**
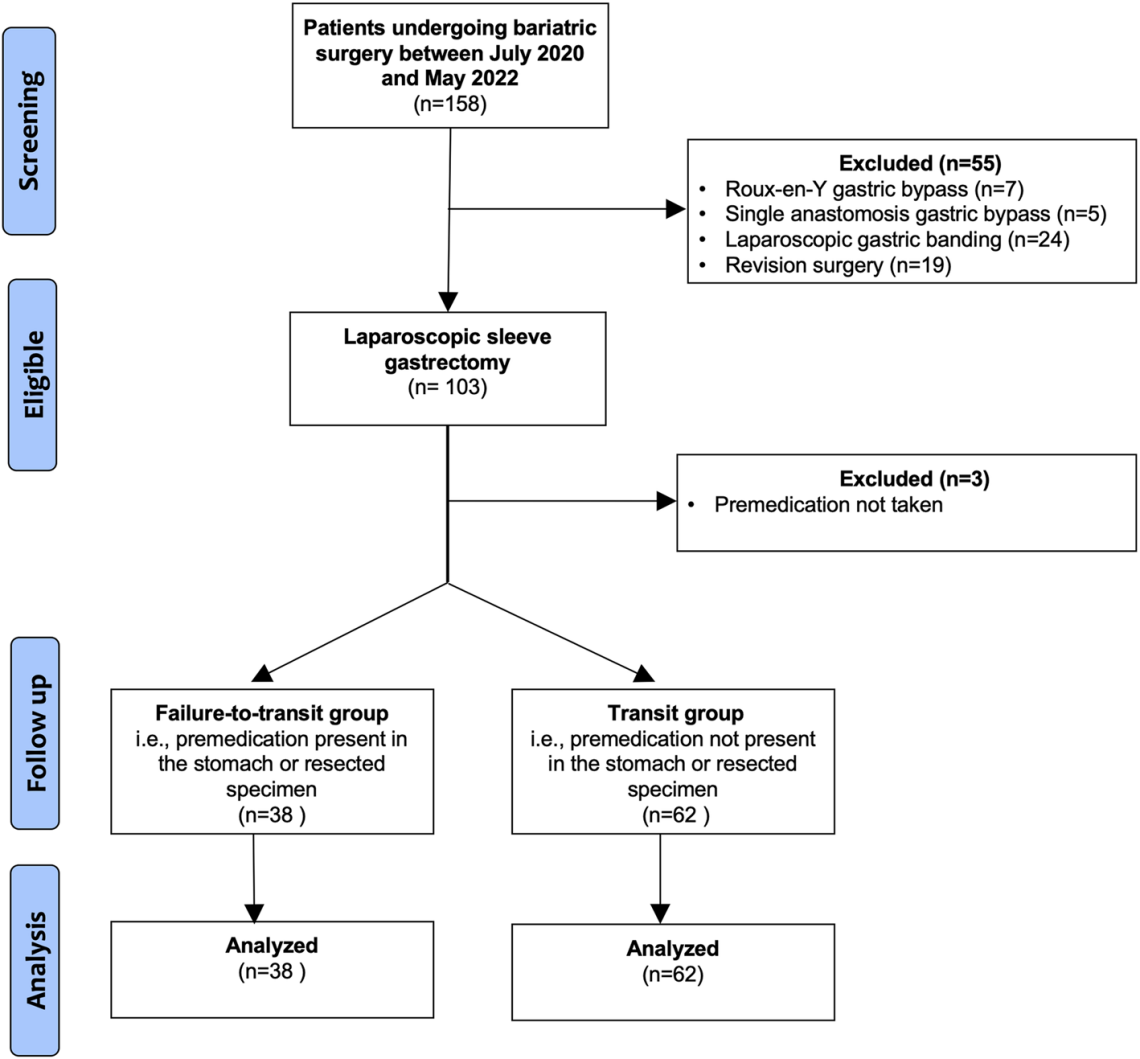
Flow diagram

A total of 158 patients underwent bariatric surgery between July 2020 and May 2022. Patients who underwent Roux-en-Y gastric bypass (*n* = 7), single anastomosis gastric bypass (*n* = 5), laparoscopic gastric banding (*n* = 24) and revision surgery (*n* = 12) were excluded. In total 103 patients underwent laparoscopic sleeve gastrectomy, of which three did not take premedication (Fig. 2). There were no missing data, and all patients were included in the analysis.

A total of 100 patients (100%) received tapentadol slow release and 89 patients (89%) received netupitant/palonosetron. The mean (SD) age was 39.9 (11.4) years. Sixty-six patients (66%) were female. The median (IQR) body mass index (BMI) was 42 kg/m^2^ (37:47). Ninety-nine patients (99%) were morbidly obese (body mass index > 35 kg/m^2^ and 17 patients (17%) were Class V obesity (BMI > 50 kg/m^2^). Type 2 diabetes mellitus was present in 17 patients (17%). No patient had type 1 diabetes mellitus or a known hiatus hernia. None of the patients had any documented history of ischemic heart disease, peripheral vascular disease, or stroke. The complete de-identified database is presented in Additional file 1.

The baseline characteristics of patients in which the tablet was present (Failure-to-Transit group) or absent (Transit group) in the resection specimen are shown in Table 1. No statistically significant differences were observed between the Failure-to-Transit and Transit groups with regards to age, sex, body mass index, or presence or absence of diabetes. All these parameters were not correlated with the presence or absence of medications in the resected specimens, except premedication lead time (Spearman’s correlation coefficient = 0.277, *P* = 0.005).

**Table 1:** Baseline patient characteristics and information on types and number of premedications

Variables		Transit group (*N* = 62)	Failure-to-Transit group (*N* = 38)	*P* value	rho (*P* value)
Age (years)		35 (29.8 ‒ 42.3) [18:64]	41 (33.8 ‒ 49.0) [22:71]	0.064	0.186 (0.064)
Female gender		31 (81.6)	48 (77.4)	0.620	-0.05 (0.624)
Weight (kg)		128.5 (112.0 ‒ 141.5) [74.6:243]	116.5 (100.0 ‒ 137.3) [81:186]	0.104	-0.163 (0.104)
Height (cm)		1.7 (1.7 ‒ 1.7) [1.5:1.9]	1.7 (1.6 ‒ 1.7) [1.5:1.9]	0.326	-0.099 (0.329)
Body mass index (kg/m^2^)		43.6 (40.5 ‒ 47.9) [27.4:82.1]	40.5 (35.0 ‒ 46.6) [28.4:62.9]	0.124	-0.155 (0.125)
Diabetes or Haemoglobin A1c level > 5.7%		7 (18.4)	10 (16.1)	0.767	-0.03 (0.770)
Premedication lead time (min)		80 (57.8 ‒ 140.0) [41:238]	119.5 (85.0 ‒ 171.3) [30:365]	0.006*	0.277 (0.005)*
**Type of premedication**					
Tapentadol premedication tablets visualized in the gastric remnant	1 tablet	11 (28.9)	27 (43.5)	0.308	-0.148 (0.147)
	2 tablets	27 (71.1)	34 (54.8)		
	4 tablets	0 (0.0)	1 (1.6)		
Netupitant/palonosetron premedication capsules visualized in the gastric remnant	None	6 (15.8)	5 (8.1)	0.238	0.12 (0.235)
	1 capsule	32 (84.2)	57 (91.9)		
**Total number of premedications administered**					
Total premedications administered	2 premedications	17 (44.7)	32 (51.6)	0.751	-0.058 (0.568)
	3 premedications	21 (55.3)	29 (46.8)		
	5 premedications	0 (0.0)	1 (1.6)		

Data are presented as median (Q1 ‒ Q3) [Min:Max] or number (%). *: *P* value < 0.05. Statistical analysis was performed using Mann-Whitney Test for continuous data, Chi-squared test for contingency tables, Cochran–Armitage test for trend for contingency table having ordered categorical data. rho: Spearman’s correlation coefficient

**Fig. 3:**
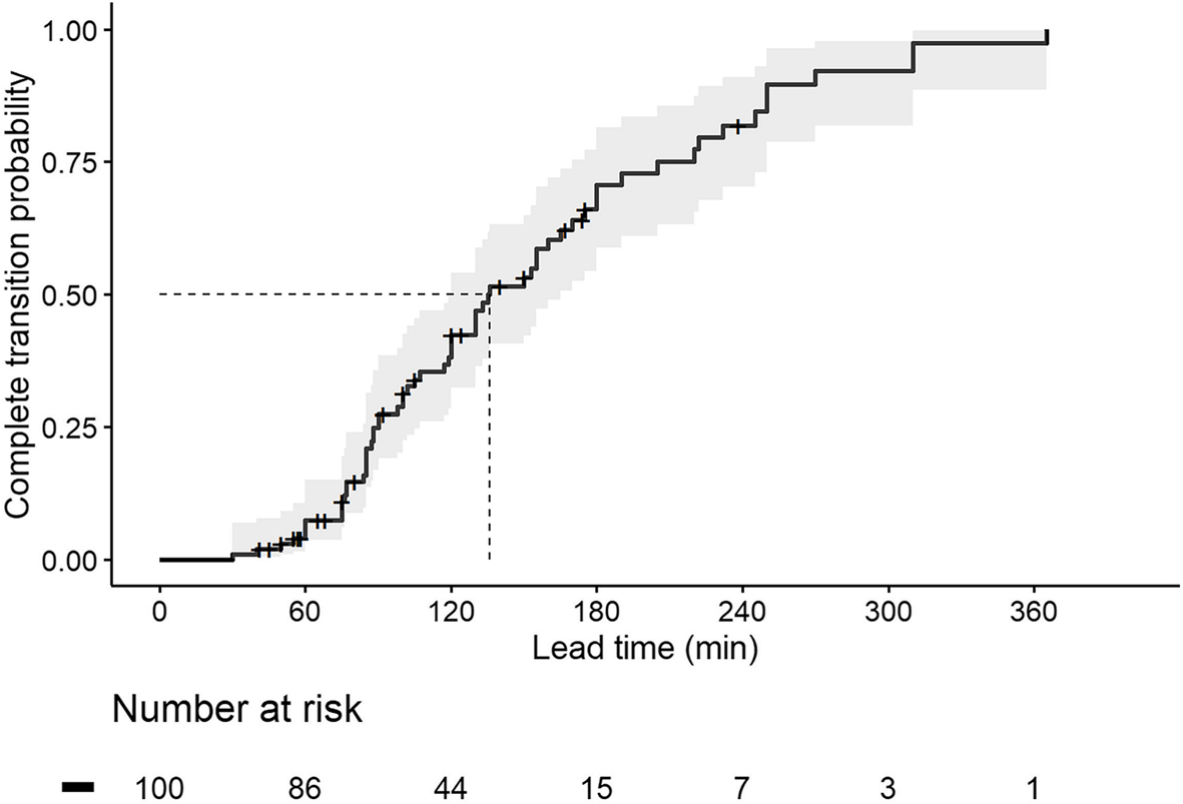
Kaplan-Meier curve of premedicated tablet transiting beyond the stomach i.e., not seen in the resection specimen or stomach. Lead time is the time from premedication administration to specimen resection. A shadowed area indicates the 95% confidence interval of the predicted probability. The dashed line indicates the median complete transit time (136 min, 95% CI: 120 ‒ 165 min). Each step-up indicates the case of patients where the premedication was not observed in the specimen; each black cross indicates a case where the premedication was observed in the specimen

**Fig. 4:**
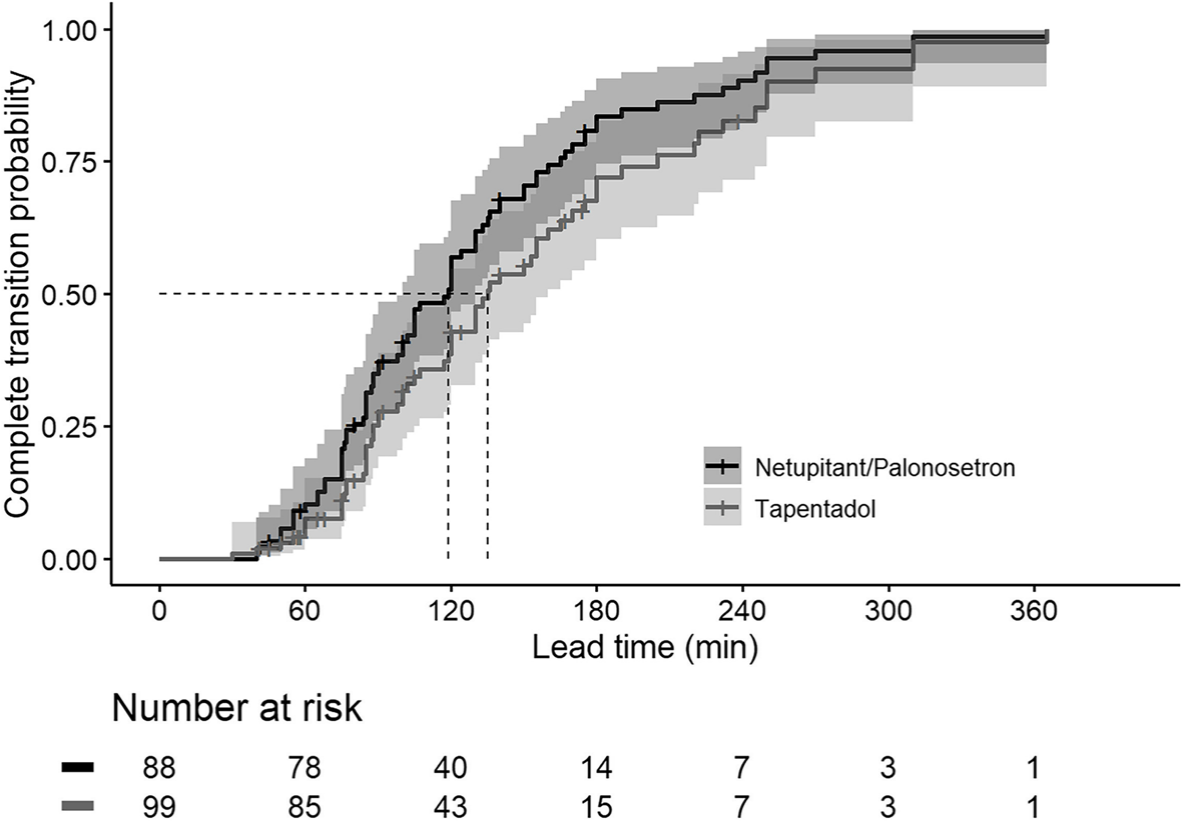
Comparative Kaplan-Meier curves of each premedicated tablet or capsule transiting beyond the stomach. Lead time is the time from premedication administration to specimen resection. A shadowed area indicates the 95% confidence interval of the predicted probability. The dashed line indicates the median complete transit time (135 min [95% CI: 120 min ‒ 165 min] for tapentadol, 119 min [95% CI: 100 min ‒ 133 min] for netupitant/palonosetron). Each step-up indicates the case of patients where the premedication was not observed in the specimen; each cross indicates a case where the premedication was observed in the specimen

 The median (Q1‒ Q3 [min:max]) premedication lead time was 80 min (57.8 ‒ 140.0 [41:238]) in the Failure-to-Transit group and 119.5 min (85.0 ‒ 171.3 [30:365]) in the Transit group; *P* = 0.006. Detailed information on the number of premedication tablets administered and the presence or absence of medications in the resected specimens are presented in Table 2. The estimated lead times for patients whom premedication transited beyond the stomach are shown in Table 3. The estimated median complete transit time of premedications was 136 min (95% CI: 120 ‒ 170 min), and the estimated 80% complete transit time was 232 min (95% CI: 180 ‒ 310 min) by the Kaplan-Meier survival analysis (Fig. 3). We also evaluated each premedication’s survival curves; the estimated median and 80% complete transit time (95% CI) were 135 min (120 ‒ 165 min) and 222 min (180 ‒ 310 min) for Tapentadol, 119 min (100 ‒ 133 min) and 175 min (155 ‒ 238 min) for Netupitant/palonosetron. The estimated complete transit curves of premedications were significantly different (*P* = 0.049), but the estimated 95% CIs overlapped considerably with each other (Fig. 4).

**Table 2:** Description of the premedications visualized in the resected specimen

Variables		Transit group (*N* = 62)	Failure-to-Transit group (*N* = 38)	*P* value
Number of tablets or capsules in specimen	0 tablet/capsule	62 (100)	0 (0)	< 0.001*
	0.5 tablet/capsule	0 (0)	2 (5.3)	
	1 tablet/capsule	0 (0)	15 (39.5)	
	2 tablets/capsules	0 (0)	17 (44.7)	
	2.5 tablets/capsules	0 (0)	1 (2.6)	
	3 tablets/capsules	0 (0)	3 (7.9)	
State of tablets in specimen	No tablets/capsule seen	62 (100)		< 0.001*
	A portion of the tablets/capsule visualized	0 (0)	30 (78.9)	
	The full tablet or capsule visualized	0 (0)	8 (21.1)	

Data are presented as number (%). *: *P* *<* 0.05 with Cochran–Armitage test for trend.

**Table 3:** The estimated lead times for patients whom premedication transited beyond the stomach

Proportions (%) of patients with transit of medication beyond the stomach	Estimated lead time (95%CI)
80	232 (180 ‒ 310)
85	250 (220 ‒ NA^a^)
90	270 (245 ‒ NA^a^)
95	310 (250 ‒ NA^a^)
100	365 (NA ‒ NA^a^)

^a^The upper value of the confidence interval for 85%–95% proportions cannot be calculated because of the sparse data in the last part of the lead time. Only four patients remained without determination (transit or non-transit of premedication) after 300 minutes

## Discussion

In this observational study we found that oral analgesia and antiemetic medication administered preoperatively did not transit beyond the stomach in 38% of patients of patients undergoing laparoscopic sleeve gastrectomy. This negated the potential pharmacological benefits of premedication in preventing postoperative nausea and vomiting and improving perioperative analgesia. We observed no significant associations between medication transit beyond the stomach and age, sex, diabetes, or body mass index. Moreover, we found that oral premedication should be administered at least 4 h before surgery to ensure transition beyond the stomach.

### Type of medication and drug absorption site

Both tapentadol and netupitant/palonosetron have been reported to be detected at 30 min post administration (implying transit beyond the stomach and into the small intestine), with peak plasma levels measured between 3 and 6 h post ingestion [16–18]. We found that the lead time required for premedication to transition beyond the stomach in 80% of the patients was approximately 4 h. Importantly, we found that the true mean time, that is, the 95% confidence interval, ranging between 3 and 6 h. The lower range of this confidence limit is substantially longer than the absorption times reported for tapentadol and netupitant/palonosetron [16–19].

There are a few plausible mechanisms explaining our findings of a long premedication gastric transit time. First, food ingested several hours after medication accelerates tablet transit through the terminal ileum and shortens the transit through the small intestine [20]. Given that all patients in our study were required to fast for solid foods for at least 6 h, the inability to eat may have affected gastric transit times. Second, the transit of medications through the stomach has been shown to be dependent on the density and size of tablets. The 50 and 100 mg slow-release tapentadol tablets administered to patients in this study were 6.5 mm x 15 mm in dimension, and the netupitant/palonosetron tablets had a length of 21.7 mm. A study of gastrointestinal transit of mini-tablet controlled release oral dosage forms in fasted human volunteers compared the gastrointestinal transit of small diameter (3.2 mm), and larger diameter (6.6 and 12.2 mm) tablets all of different densities and found that the only tablet system to enter the cecum within the time limit of the study was the 12.2-mm tablets [21]. In a similar study, irrespective of tablet density, smaller tables i.e. 6.6-mm had longer gastric emptying times than the larger one of 12.0-mm [22].

Third, we found no significant association between obesity and the gastric transit time. Some studies have reported that obesity reduces esophageal and gastric motility through neuroendocrine dysregulation [23, 24]. In another study, bariatric patients were shown to have an inverse relationship between weight and gastric emptying compared to non-overweight populations [25]. This finding was observed despite comorbid conditions, such as diabetes, which has also been shown to delay gastric emptying due to autonomic neuropathy affecting the stomach [26]. However, the literature on this topic is disparate. In a review, Knibble et al. reported that drug absorption in obese populations remains largely unaltered [27].

Fourth, it has been shown that type 2 diabetes can reduce gastric emptying [28]. We found no association between diabetes or high hemoglobin A1c levels and gastric emptying, possibly because our median population age was only 39 years. Accordingly, patients with diabetes may not have had the time to develop gastroparesis and autonomic neuropathy. The 10-year incidence of symptomatic gastroparesis in patients with type 2 diabetes is approximately 1% with a predominant risk factor being the presence of diabetes over 10 years [26, 29]. Finally, other causes of autonomic neuropathy of the stomach include previous abdominal or esophageal surgery, amyloidosis, autoimmune or connective tissue disorders, or nervous system diseases such as Parkinson’s disease or multiple sclerosis, none of which were present in our patient cohort.

### Other literature regarding administration time of premedication

Few studies have examined the optimal timing of premedication in the bariatric population. Varbanova et al. [30] reviewed the use of midazolam, melatonin, pregabalin, and gabapentin as premedications. The authors suggest premedication lead times of half an hour, 50 min, 1.5 h, and 2–3 h respectively. The variation in dose timing is due to the difference in time to peak effect; however, having multiple timings for different premedications may not be pragmatic in a busy surgical setting. Pawlik et al. [31] evaluated the effect of premedication with clonidine on patients with OSA, with reported lead times between 2 and 12 h prior to surgery. Here, the administration of the medication via two doses ensured complete absorption of the first dose, with an unknown transit of medication of the second. This supports our finding that longer premedication lead times may be required to ensure complete transit and drug action.

### Strengths and limitations

This study had several limitations that are intrinsic to its retrospective design. These findings may not be generalizable to other types of bariatric and non-bariatric surgery or to non-obese patients. Our findings cannot be generalized to other types of premedications administered in the perioperative period. The small sample size limits the systematic evaluation of clinically meaningful outcomes such as postoperative nausea and vomiting, pain scores, and length of hospital stay. No plasma levels of tapentadol and netupitant/palonosetron were measured; therefore, the primary outcome of observing the premedication in the stomach or resected specimen provides insight into the pharmacokinetic properties of both medications, especially systemic absorption. Furthermore, visual identification of some tablets in the stomach was not possible because of their unrecognizable physical state. Therefore, conclusions cannot be drawn regarding the differences in gastric transit between the two medications.

Importantly, we acknowledge that we cannot exclude the presence or absence of premedication tablets or capsules in the portion of the stomach that was still in-situ post sleeve resection. This would only have been possible to assess if all patients had an on-table gastroscopy, which patients did not consent to. Accordingly, our findings may be an underestimation the true failure-to-transit rate. We argue however, that any retained material in the stomach would normally lie in the lateral stomach or fundus rather than along the lesser curve, therefore more likely to be in the resected specimen.

Our study has several strengths. No previous studies have investigated tablet or capsule transit using direct observation. Our findings provide accurate data regarding the medication transit time and pilot data for the design of future interventional studies evaluating techniques to improve gastric transit times in this patient cohort.

## Conclusion

Oral analgesia and antiemetic medication administered preoperatively did not transit beyond the stomach in 38% of patients undergoing laparoscopic sleeve gastrectomy, and no significant associations were observed between medication transit time and age, sex, diabetes, or body mass index. Our findings suggest that in this setting, the combination of slow release tapentadol and netupitant/palonosetron should be administered at least 4 h before surgery to ensure transition beyond the stomach. A prospective randomized trial is warranted to further investigate techniques or pharmacological strategies to improve gastric transit times in patients receiving premedication before bariatric surgery. Future enhanced recovery after bariatric surgery guidelines may benefit from the standardization of premedication lead times to facilitate increased premedication absorption.

### Supplementary Information

**Additional file 1.** Oral Premedication in Sleeve Gastrectomy Audit

## Data Availability

All data generated or analyzed during this study are included in this published article and its supplementary information files.
