# Supplementary material for: The transit of oral premedication beyond the stomach in patients undergoing laparoscopic sleeve gastrectomy: a retrospective observational multicentre study
Source: BMC Surg. 2023 Nov 4;23:335. doi: 10.1186/s12893-023-02246-6 (PMC10625241; doi:10.1186/s12893-023-02246-6)
Supplement: Supplementary file 1 — Additional file 1. Oral Premedication in Sleeve Gastrectomy Audit [file 12893_2023_2246_MOESM1_ESM.pdf]

Oral Premedication in Sleeve Gastrectomy Audit

Pat	Date	Age	Sex	Wt(kg)	Ht(m)	BMI	Diabetes	Admission	Palexia Premed	Akynzeo Premed	Anaes Start	Sleeve Start	Sleeve Finish	Premed Lead time	Tablets in Specimen	Total Tabs Given	o of Palexia prese	o of Akynzeo prene	Descriptor
1	27/7/20	32	F	100	1.72	34	N	0800	0850(1 tab)	0910	0940	1022	1035	92	2	2	1	1	
2	27/7/20	31	M	144	1.81	44	N	0830	0855(1 tab)	0855	1110	1150	1205	175	2	2	1	1	
3	9/10/20	45	M	117	1.76	38	N	0930	1015(2 tab)	NA	1230	1248	1318	153	0	2	0	NA	
4	9/10/20	31	F	131	1.64	49	N	1130	1410(2 tab)	NA	1630	1714	1723	174	1	2	1	NA	
5	9/10/20	44	F	114	1.77	36	N	1230	1445(2 tab)	NA	1750	1827	1836	222	0	2	0	NA	
6	19/10/20	38	M	137	1.81	42	N	0800	0900(1 tab)	0900	1100	1130	1141	150	0.5	2	unidentifiable	unidentifiable	1 - partial(0.5)
7	6/11/20	35	F	118	1.74	39	N	1030	1105(2tab)	1115	1215	1243	1250	98	0	3	0	0	
8	6/11/20	63	F	106	1.54	45	N	1130	1230(2tabs)	1230	1320	1355	1405	85	0	3	0	0	
9	6/11/20	51	M	104	1.67	37	N	1430	1515(2tabs)	1515	1730	1807	1820	232	0	3	0	0	
10	16/11/20	24	F	150	1.72	51	N	0730	0925(2tab)	0925	1015	1033	1045	68	1	3	1	0	
11	16/11/20	27	F	112	1.73	37	N	0800	1115(2tabs)	1100	1125	1141	1155	41	2	3	2	0	
12	4/12/20	22	F	101	1.54	43	N	0700	0805(1tab)	0805	0840	0910	0925	55	0	2	0	0	
13	4/12/20	52	F	146	1.67	52	N	0800	0925(1tab)	0925	1000	1042	1052	77	0	2	0	0	
14	4/12/20	31	M	146	1.82	44	N	1000	1105(1tab)	1105	1140	1210	1219	65	1	2	1	0	Palexia intact
15	4/12/20	39	F	145	1.63	55	N	1400	1530(1tab)	1530	1610	1647	1657	77	0	2	0	0	
16	4/12/20	48	F	85	1.59	34	N	1500	1645(1tab)	1645	1730	1812	1820	87	0	2	0	0	
17	14/12/20	30	F	109	1.55	45	N	0630	0810(2tab)	0830	0840	0908	0915	58	2.5	3	2	0.5	Akynzeo capsule partial 0.5
18	14/12/20	47	F	91	1.62	35	N	0730	0845(2tab)	0845	1000	1027	1035	102	0	3	0	0	
19	14/12/20	40	F	97	1.56	40	N	0830	1025(2tabs)	1025	1115	1140	1150	75	0	3	0	0	
20	29/1/21	46	F	110	1.62	42	Y	0715	0830(1tab)	0830	0945	1017	1030	107	0	2	0	0	
21	29/1/21	33	F	139	1.66	50	N	0815	0950(1tab)	0950	1115	1203	1210	133	0	2	0	0	
22	29/1/21	64	F	138	1.65	51	Y	0930	1115(2tabs)	1115	1251	1335	1347	140	1	3	1	0	
23	29/1/21	47	F	105	1.67	38	N	1215	1545(1tab)	1545	1645	1725	1738	100	0	2	0	0	
24	29/1/21	29	M	140	1.78	44	N	1345	1505(1tab)	1505	1800	1900	1910	245	0	2	0	0	
25	26/2/21	56	F	108	1.64	40	N	0900	1100(1tab)	1100	1240	1316	1323	136	0	2	0	0	
26	26/2/21	43	F	108	1.69	38	N	1100	1145(1tab)	1145	1400	1430	1440	165	0	2	0	0	
27	26/3/21	47	F	92	1.65	34	Y	0900	1054(1tab)	1054	1123	1210	1220	76	0	2	0	0	
28	26/3/21	24	F	94	1.59	37	N	1030	1136(1tab)	1136	1300	1335	1345	119	0	2	0	0	
29	26/3/21	55	F	147	1.66	53	N	1330	1535(1tab)	1535	1815	1915	1925	220	0	2	0	0	
30	23/4/21	29	F	138	1.56	57	N		1245(2tabs)	1245	1510	1540	1547	175	0	3	0	0	
31	23/4/21	44	F	104	1.74	34	N		1400(1tab)	1400	1625	1700	1710	180	0	2	0	0	
32	23/4/21	27	F	112	1.66	41	N		1620(1tab)	1620	1800	1840	1850	140	0.5	2	0	0.5	1 - partial(0.5)
33	3/5/21	54	F	130	1.65	48	Y	0800	0835(2tab)	0835	1045	1125	1135	170	0.00	3	0	0	
34	21/5/21	29	F	143	1.69	50	N	0930	1150(1tab)	1150	1300	1335	1344	105	1	2	1	0	
35	21/5/21	23	F	120	1.66	44	N	1530	1550(1tab)	1550	1815	1845	1854	175	1	2	1	0	
36	18/6/21	26	F	130	1.64	48	N	1300	1350(2tabs)	1350	1545	1620	1632	150	2	3	2	0	
37	19/6/21	42	M	178	1.88	50	N	0900	1045(2tabs)	1045	1100	1200	1215	75	2	3	2	0	
38	19/6/21	52	M	123	1.8	38	Y	1000	1135(2tabs)	1135	1315	1350	1400	135	0	3	0	0	
39	19/6/21	49	F	122	1.67	44	Y	1100	1340(2tabs)	1340	1500	1525	1535	105	2	3	2	0	
40	16/7/21	24	F	114	1.67	41	N	0730	0845(1tab)	0845	0945	1015	1025	90	0	2	0	0	
41	16/7/21	37	F	131	1.67	47	N	0900	0930(1tab)	0930	1120	1150	1158	140	1	2	1	0	
42	17/7/21	35	F	128	1.66	46	N	0800	0845(2tab)	0845	0930	1013	1030	88	0	3	0	0	
43	24/7/21	34	M	138	1.75	45	N	0730	0845(2tab)	0845	0940	1013	1023	88	0	3	0	0	
44	24/7/21	60	F	88	1.67	32	N	0900	1035(2tabs)	1035	1415	1545	1600	310	0	3	0	0	
45	26/7/21	26	F	155	1.8	48	Y	0730	0825(2tabs)	0825	0835	0910	0920	45	3	3	2	1	
46	7/8/21	27	F	108	1.59	43	N	0800	0840(2 tabs)	0840	0940	1005	1013	85	0	3	0	0	
47	7/8/21	49	F	103	1.67	37	N	0900	0958(2tabs)	0958	1050	1128	1138	90	0	3	0	0	
48	7/8/21	43	F	143	1.8	44	N	1200	1315(2tabs)	1315	1500	1602	1610	167	2	3	2	0	
49	7/8/21	29	F	127	1.69	44	N	1200	1342(2tabs)	1342	1710	1740	1750	238	2	3	2	0	
50	13/8/21	32	F	87	1.65	32	N	0630	0758(2tabs)	NA	0820	0855	0905	57	2	2	2	NA	
51	13/8/21	35	F	152	1.7	53	N	0900	1115(1tab)	1115	1545	1625	1635	310	0	2	0	0	
52	13/8/21	56	M	119	1.72	40	N	1130	1145(1tab)	1145	1705	1750	1758	365	0	2	0	0	
53	13/8/21	29	M	134	1.76	43	N	1300	1500(1tab)	1500	1840	1910	1920	250	0	2	0	0	
54	21/8/21	58	F	128	1.66	46	N	0800	0825(2tabs)	0825	1000	1029	1039	124	2	3	2	0	
55	23/8/21	31	F	243	1.72	82	N	0730	0820(2tabs)	0820	0925	1020	1030	120	2	3	2	0	
56	23/8/21	24	F	100	1.62	38	N	0800	0853(2 tabs)	0853	1130	1218	1228	205	0	3	0	0	
57	28/8/21	43	F	97	1.67	35	N	0830	1000(2tabs)	1000	1130	1157	1206	117	0	3	0	0	
58	28/8/21	34	F	100	1.7	35	N	0930	1035(2tabs)	1035	1240	1310	1322	155	0	3	0	0	
59	28/8/21	49	F	86	1.65	32	N	1200	1230(2tabs)	1230	1410	1440	1448	130	0	3	0	0	
60	28/8/21	27	F	186	1.72	63	N	1300	1350(2tabs)	1350	1530	1600	1610	130	0	3	0	0	
61	10/9/21	40	F	165	1.71	56	Y	1000	1215(1tab)	1215	1225	1305	1315	50	0	2	0	0	
62	10/9/21	37	F	99	1.66	36	N	1330	1645(1tab)	1645	1700	1725	1733	40	0	2	0	0	
63	11/9/21	37	F	136	1.78	43	N	0900	1015(2 tab)	1015	1030	1100	1008	45	1	3	1	0	
64	11/9/21	42	F	129	1.7	45	N	1200	1230(2tabs)	1230	1340	1410	1418	100	3	3	2	1	
65	20/9/21	58	F	115	1.67	41	Y	0730	0845(2tab)	0910	0925	0953	1000	68	1	3	1	0	
66	20/9/21	39	M	132	1.83	39	N	0830	1020(2tabs)	1000	1100	1135	1143	75	2	3	2	0	
67	8/10/21	23	F	132	1.71	45	N	0800	0930(2tabs)	0930	1330	1400	1410	270	0	3	0	0	
68	8/10/21	71	M	147	1.77	47	N	1330	1700(1tab)	1700	1820	1900	1910	120	0	2	0	0	
69	8/10/21	28	M	118	1.85	34	N	1430	1800(1tab)	1800	2000	2035	2043	155	0	2	0	0	
70	15/11/21	39	F	131	1.65	48	N	0730	0805(2tabs)	0805	1000	1035	1043	150	0	3	0	0	
71	15/11/21	40	F	130	1.7	45	Y	0800	0908	0908	1115	1148	1158	160	0	3	0	0	
72	7/1/22	35	F	74.6	1.65	27	N	0730	0930(2tabs)	NA	0940	1015	1025	45	1	2	1	NA	
73	7/1/22	18	F	113	1.67	41	N	0930	1032(2 tabs)	NA	1110	1147	1157	75	1	2	1	NA	
74	8/1/22	40	F	133	1.66	48	N	0930	1010(2 tabs)	1010	1030	1100	1110	50	2	3	2	0	
75	8/1/22	50	M	116	1.82	35	N	1200	1300(2 tabs)	1300	1630	1710	1720	250	0	3	0	0	
76	7/3/22	42	F	115	1.69	40	N	0700	0800(2 tabs)	0800	0815	0855	0907	55	2	3	2	0	
77	7/3/22	45	M	127	1.84	38	N	0700	0905(2 tabs)	0905	0945	1030	1038	85	0	3	0	0	
78	7/3/22	54	F	89	1.6	35	Y	0900	1030(2 tabs)	1030	1115	1150	1200	80	3	3	2	1	
79	7/3/22	29	F	89	1.62	34	Y	1300	1630	1630	1715	1745	1755	75	0	3	0	0	
80	19/3/22	35	F	83	1.71	28	N	0730	0910(2tabs)	0910	1030	1110	1122	120	0	3	0	0	
81	19/3/22	39	M	150	1.92	41	N	1200	1330(2tabs)	1330	1530	1630	1645	180	0	3	0	0	
82	25/3/22	48	M	141	1.71	48	Y	1000	1205(1tab)	1205	1230	1325	1335	80	1	2	1	0	
83	25/3/22	50	F	137	1.62	52	N	1130	1255(1tab)	1255	1435	1535	1550	100	0	2	0	0	
84	25/3/22	34	F	138	1.62	53	N	1300	1400(1tab)	1400	1640	1710	1717	190	0	2	0	0	
85	25/3/22	45	F	116	1.87	33	N	1400	1700(1tab)	1700	1800	1825	1836	85	0	2	0	0	
86	30/4/22	64	F	81	1.67	29	N	0615	0730(2tab)	0	0730	0800	0810	30	0	2	0	NA	
87	30/4/22	49	F	102	1.56	42	N	0730	0925(2tab)	0	1000	1025	1035	60	0	2	0	NA	
88	30/4/22	38	F	86	1.6	34	Y	0930	1110(2tabs)	0	1120	1210	1220	60	0	2	0	NA	
89	30/4/22	30	M	155	1.89	43	N	1100	1210(2tabs)	0	1250	1325	1335	75	2	2	2	NA	
90	2/5/22	66	F	87	1.61	34	N	0800	0920(2tabs)	0920	0945	1044	1050	84	0	3	0	0	
91	2/5/22	30	F	127	1.75	41	N	0900	1040(4tabs)	1040	1120	1155	1202	75	0	5	0	0	
92	2/5/22	34	F	115	1.63	43	N	1200	1250(2tabs)	1250	1300	1355	1406	65	2	3	2	0	
93	2/5/22	27	F	128	1.71	44	N	1330	1405(2tabs)	1405	1500	1550	1600	105	0	3	0	0	
94	20/5/22	50	F	100	1.54	42	Y	0630	0800(2 tabs)	0	0810	0850	0900	50	2	2	2	NA	
95	20/5/22	41	F	89	1.63	33	N	1000	1125(1)	1125	1245	1325	1329	120	1	2	1	0	Spilt into peritoneal cavity
96	20/5/22	41	M	130	1.75	42	Y	1130	1245(1)	1245	1400	1445	1455	120	0	2	0	0	
97	20/5/22	51	F	112	1.66	41	N	1300	1420(1)	1420	1535	1605	1612	105	1	2	1	0	
98	20/5/22	47	M	145	1.69	51	N	1400	1530(1tab)	1530	1650	1740	1746	130	0	2	0	0	
99	30/5/22	35	F	113	1.72	38	N	0630	0815(2tab)	0815	0835	0910	0918	55	1	3	1	0	
100	30/5/22	41	F	150	1.67	54	Y	0830	0955(2tab)	0955	1015	1055	1105	60	0	3	0	0	
